# Comparative physiology and transcriptome analysis reveals that chloroplast development influences silver-white leaf color formation in *Hydrangea macrophylla* var. *maculata*

**DOI:** 10.1186/s12870-022-03727-1

**Published:** 2022-07-16

**Authors:** Xiangyu Qi, Shuangshuang Chen, Huadi Wang, Jing Feng, Huijie Chen, Ziyi Qin, Yanming Deng

**Affiliations:** 1 Jiangsu Key Laboratory for Horticultural Crop Genetic Improvement, Institute of Leisure Agriculture, Jiangsu Academy of Agricultural Sciences, Nanjing, Jiangsu, 210014 China; 2 School of Life Sciences, Jiangsu University, Zhenjiang, Jiangsu, 212013 China

**Keywords:** *Hydrangea macrophylla*, Leaf color, Chlorophyll, Transcriptome, Chloroplast development

## Abstract

**Background:**

*Hydrangea macrophylla* var. *Maculata* ‘Yinbianxiuqiu’ (YB) is an excellent plant species with beautiful flowers and leaves with silvery white edges. However, there are few reports on its leaf color characteristics and color formation mechanism.

**Results:**

The present study compared the phenotypic, physiological and transcriptomic differences between YB and a full-green leaf mutant (YM) obtained from YB. The results showed that YB and YM had similar genetic backgrounds, but photosynthesis was reduced in YB. The contents of pigments were significantly decreased at the edges of YB leaves compared to YM leaves. The ultrastructure of chloroplasts in the YB leaves was irregular. Transcriptome profiling identified 7,023 differentially expressed genes between YB and YM. The expression levels of genes involved in photosynthesis, chloroplast development and division were different between YB and YM. Quantitative real-time PCR showed that the expression trends were generally consistent with the transcriptome data.

**Conclusions:**

Taken together, the formation of the silvery white leaf color of *H. macrophylla* var. *maculata* was primarily due to the abnormal development of chloroplasts. This study facilitates the molecular function analysis of key genes involved in chloroplast development and provides new insights into the molecular mechanisms involved in leaf coloration in *H. macrophylla*.

**Supplementary Information:**

The online version contains supplementary material available at 10.1186/s12870-022-03727-1.

## Background

Hydrangea (*Hydrangea macrophylla* (Thunb.) Ser.) is a deciduous species belonging to the family Saxifragaceae. Hydrangea is a popular ornamental plant species in Asia, America and Europe [1, 2]. The sepal color of hydrangea plants changes from white to pink, red, purple and blue when cultured in soils with different pH values and Al^3+^ contents [3]. Famous for its charming large and multicolored flowers, hydrangea is extensively used as potted, bouquet and landscape plants.

Chloroplasts produce carotenoids and chlorophyll, and chlorophyll is the main pigment component in green leaves [4]. Many studies focused on the biosynthesis and degradation of chlorophyll. For example, the chlorophyll biosynthesis pathway in *Arabidopsis thaliana* starts from glutamyl-tRNA to chlorophylls *a* and *b*, and 27 genes encoding 15 enzymes for all 15 steps were identified [5]. Four enzymes are involved in the chlorophyll degradation pathway from chlorophyll *b* to nonfluorescent chlorophyll catabolites [6]. The silencing of *HrHEMA* (glutamyl-tRNA reductase) and *HrCAO* (chlorophyllide an oxygenase) genes significantly affected the structure of the chloroplast and resulted in a change in leaf color [7]. Virus-induced gene silencing of *CHLI* (magnesium-chelatase I subunit) reduced chlorophyll content and altered chloroplast function, which led to abnormal chloroplast structure in peas [8]. Impaired function of *NYC1* (*NON-YELLOW COLORING1*) or *NOL* (*NYC1-like*) resulted in a stay-green phenotype in rice [9, 10].

As the site of photosynthesis, chloroplasts consist of the chloroplast membrane, thylakoid and matrix [11]. The number and distribution of chloroplasts in the tissue primarily influence leaf color. Previous studies showed that the altered expression of genes related to chloroplasts affected the biogenesis of chloroplasts [4, 7, 12]. The disruption of chloroplast assembly may lead to abnormal leaf color [13–15]. Variation in leaf color is one of the most common phenomena in higher plant species. Leaf color mutations were identified in various green plants, such as cotton [16], wheat [17], *Anthurium andraeanum* [13], birch [18] and *Hosta plantaginea* [7]. Leaf mutants help reveal the molecular mechanisms of leaf color formation and have received increasing attention. Changes in the expression levels of key genes involved in chloroplast development and division generally result in leaf color mutations [19]. For example, maize pentatricopeptide repeat 4 (PPR4) is necessary for the normal development of chloroplasts by associating with plastid *rps12* pre-mRNA and splicing in trans [20]. The *Golden 2-like* (*GLK*) genes play a positive role in the regulation of chloroplast development [21], and *Arabidopsis glk1-glk2* double mutants showed a pale green phenotype with a lack of chloroplast thylakoid membranes and grana [22, 23]. The gene family *Accumulation and Replication of Chloroplasts* (*ARC*), *ARC3*, *ARC5* and *ARC6* regulate the division of chloroplasts [24–26]. *OscpSRP43* (chloroplast signal recognition particle 43) is required for the normal development of chloroplasts in rice, and the color mutation exhibited a distinct yellow-green leaf phenotype with impaired chloroplasts [27].

*H. macrophylla* var. *maculata* ‘Yinbianxiuqiu’ (YB) is an excellent variety with beautiful flowers and leaves with silvery white edges. A mutant with full-green leaves (YM) was obtained from one YB plant. YM and YB differed only in leaf color. However, the mechanism leading to leaf color variation in YB is not clear. Therefore, the present study compared the phenotypic and physiological characteristics of YB and YM. Leaf transcriptomes from YB and YM plants were sequenced, and genes involved in photosynthesis, chloroplast development and division and chlorophyll biosynthesis and degradation were identified. The expression of genes involved in chloroplast development and division was validated using quantitative real-time PCR (qRT–PCR). The present study elucidated the molecular mechanisms that regulate leaf color formation in *H. macrophylla* to provide a foundation for breeding hydrangea varieties with ornamental leaves.

## Results

### Phenotypic and sequence-related amplified polymorphism (SRAP) analyses

**Fig. 1:**
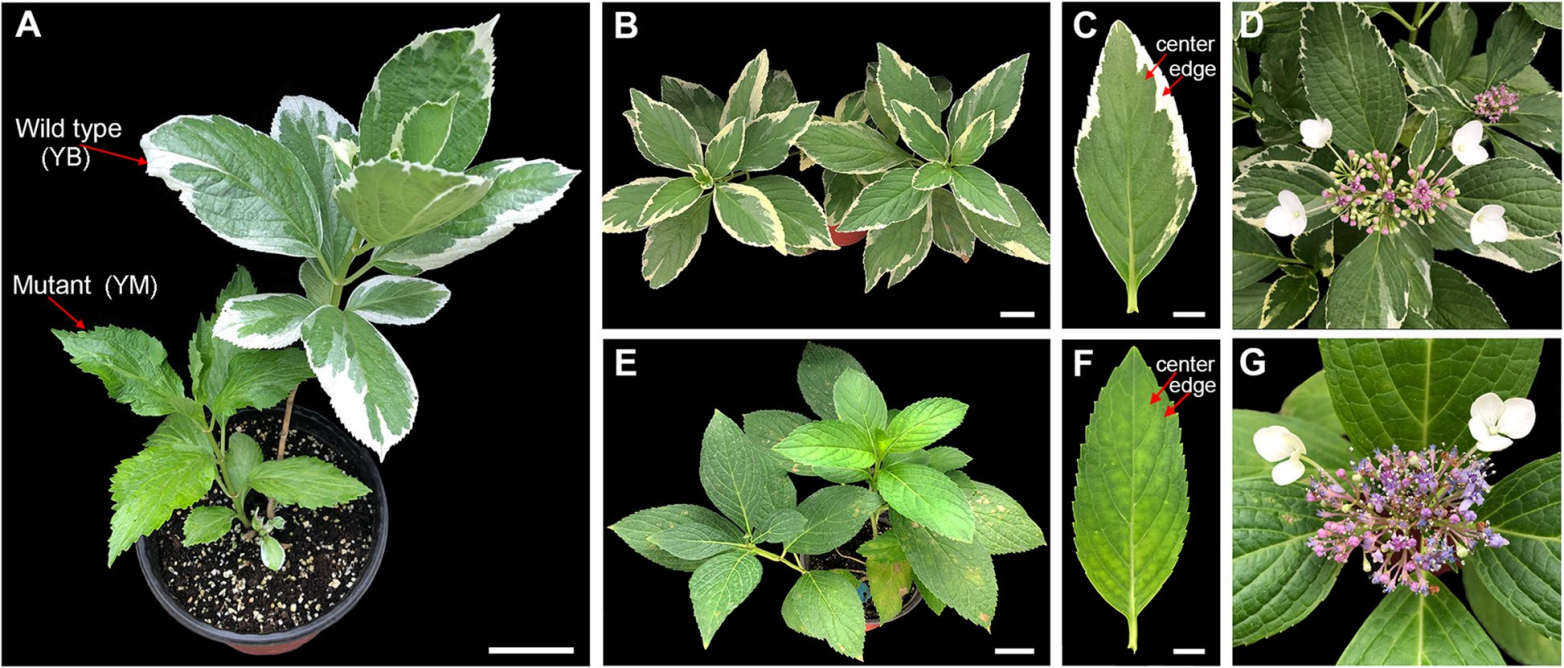
Phenotypes of *H. macrophylla* var. *maculata* (**YB**) and full-green leaf mutant (**YM**). (**A**) YM was a bud mutation derived from YB. Bar: 5 cm. (**B**) YB plant. Bar: 5 cm. (**C**) Leaf of YB. Bar: 1 cm. (**D**) Flower of YB. (**E**) YM plant. Bar: 5 cm. (**F**) Leaf of YM. Bar: 1 cm. (**G**) Flower of YM. center: central position of leaf; edge: edge position of leaf

A full-green leaf mutant (YM) was obtained from YB (Fig. 1A). After years of vegetative propagation, the full-green leaves of YM were stable and bloomed normally (Fig. 1E, F, G).

A total of 136 SRAP primers amplified 1,519 fragments from YB and 1,518 fragments from YM (Fig. S1). Most primer pairs amplified the same fragments from YB and YM, except three primer pairs, M6/E18, M13/E16 and M16/E11. Primer pair M6/E18 amplified three fragments in YB and two in YM. Primer pair M13/E16 amplified ten fragments in YB and nine in YM, and primer pair M16/E11 amplified eight fragments in YB and nine in YM. These results indicated that YB and YM had a similar genetic background.

### Photosynthesis and photosynthetic pigments content

**Fig. 2:**
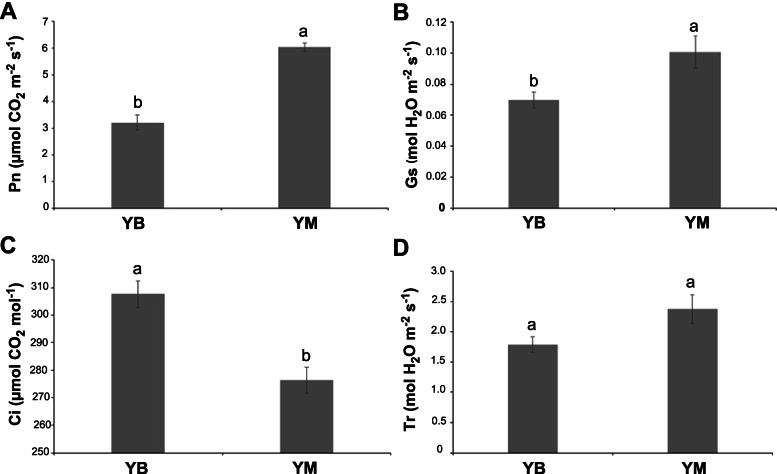
Photosynthetic parameters of *H. macrophylla* var. *maculata* (**YB**) and full-green leaf mutant (**YM**). (**A**) Net photosynthetic rate (Pn). (**B**) Stomatal conductance (Gs). (**C**) Intercellular CO_2_ concentration (Ci). (**D**) Transpiration rate (Tr)

The net photosynthetic rate (Pn) and stomatal conductance (Gs) values of YB and YM varied significantly, and both values were higher in YM (Fig. 2A, B). The intercellular CO_2_ concentration (Ci) values also varied significantly between YB and YM, and the Ci value of YB was higher than YM (Fig. 2C). However, the transpiration rate (Tr) values showed no significant variation between YB and YM (Fig. 2D). These results suggested that the plant photosynthesis capacity differed between YB and YM, and YM capacity was higher than YB.

**Fig. 3:**
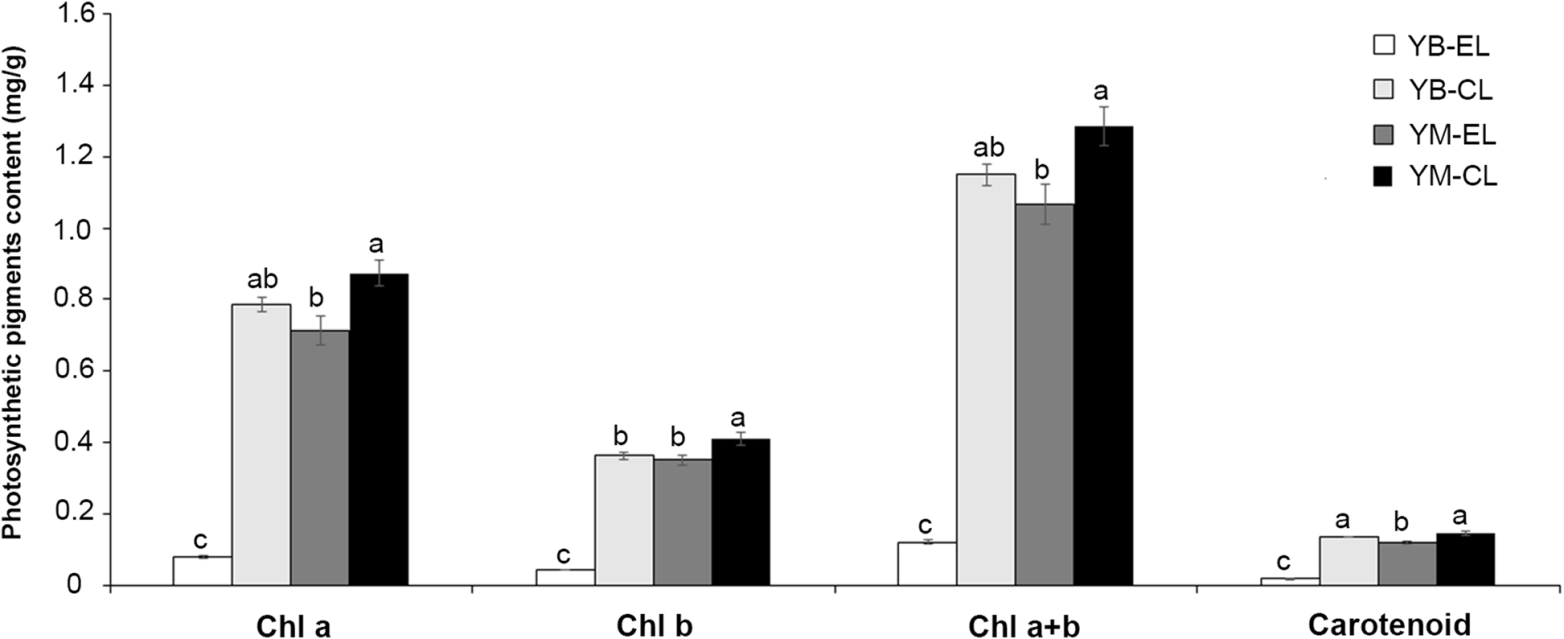
The content of photosynthetic pigments in *H. macrophylla* var. *maculata* (**YB**) and full-green leaf mutant (**YM**). EL: edged leaf; CL: central leaf

The chlorophyll and carotenoid contents were significantly different between YB and YM (Fig. 3). The highest contents of chlorophyll a, chlorophyll b and chlorophyll a + b were detected in the central position of the YM leaf, and the lowest contents were detected in the edge of the YB leaf. The highest carotenoid contents were measured in the central position of YB and YM leaves, and the lowest carotenoid contents were measured in the edges of YB leaves. These results indicated that the abnormal leaf color of YB closely correlated with the change in pigment contents.

### Chloroplast ultrastructure

**Fig. 4:**
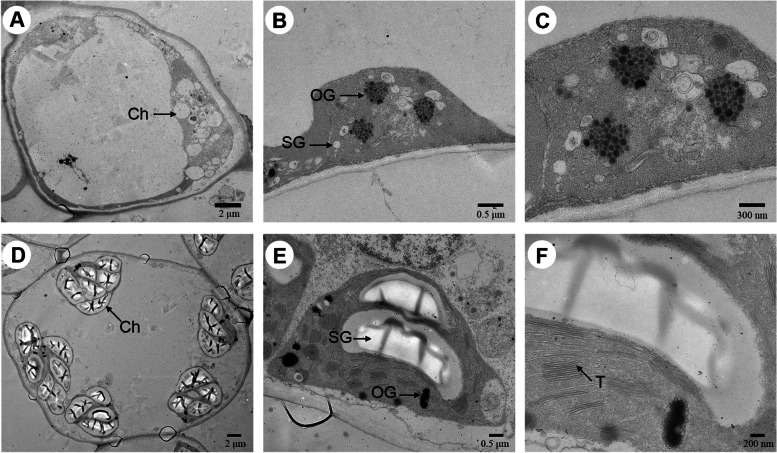
Chloroplast ultrastructure of *H. macrophylla* var. *maculata* (**YB**) and full-green leaf mutant (**YM**). (**A-C**) Chloroplast ultrastructure of YB. In (**A**), bar: 2 μm; (**B**), bar: 0.5 μm; (**C**), bar: 300 nm. (**D-F**) Chloroplast ultrastructure of YM. In (**D**), bar: 2 μm; (**E**), bar: 0.5 μm; (**F**), bar: 200 nm. Ch: chloroplast; OG: osmiophilic globules; SG: starch grains; T: thylakoid

The chloroplast ultrastructure was analyzed to verify the abnormal development of chloroplasts in the mesophyll cells of YB. The shape, size and number of chloroplasts were obviously different between YB and YM (Fig. 4). Chloroplasts had an intact stromal thylakoid structure in YM (Fig. 4E, F). However, the chloroplasts in YB had no inner member structures (Fig. 4B, C). The shape of the chloroplasts was elliptical or ovoid in YM (Fig. 4D) but swollen oblate or spheroidal in YB (Fig. 4A). Chloroplasts in the mesophyll cells of YM contained small starch granules and a few osmiophilic globules (Fig. 4E, F), but large starch granules and many osmiophilic globules were observed in YB (Fig. 4B, C). These results showed that the ultrastructure of chloroplasts in the YB leaves was irregular, which confirmed the abnormal chloroplast development.

### Illumina sequencing and assembly

Based on the above phenotypic and physiological characteristics, we speculated that the expression patterns of genes involved in chloroplast development and division and pigment metabolism were altered in YM plants. To test this hypothesis, the leaf edges of YB and YM were collected and sequenced to examine the mechanism of leaf color formation. The total number of raw reads per library ranged from 47,956,778 to 64,679,848, and the total number of clean reads ranged from 47,950,946 to 64,664,862 (Table S1). The proportion of clean reads and clean data was > 99.97% in each library (Table S1). A total of 123,122 unigenes with an N50 length of 1,235 bp were obtained from the *H. macrophylla* transcriptome via de novo assembly (Table 1). The unigenes had an average length of 778 bp, a median length of 453 bp, a minimum length of 201 bp, a maximum length of 15,890 bp, and a total length of 95,817,440 bp. The GC content of unigenes was 38.98% (Table 1). The length distribution of unigenes is shown in Fig. S2, and 17,998 unigenes had lengths over 1,000 bp.

**Table 1:** Statistics of unigene sequences in the *H. macrophylla* transcriptome

Item	Total length (bp)	Total number	GC content (%)	N50 (bp)	N90 (bp)	Average (bp)	Median (bp)	Min (bp)	Max (bp)
Value	95,817,440	123,122	38.98	1,235	316	778	453	201	15,890

### Gene functional annotation

All 123,122 assembled unigenes were annotated in the NR, UniProt, GO, KEGG, eggNOG and Pfam databases (Table S2). A total of 42,572 (34.58%) unigenes were matched in at least one of the these databases. There were 40,235, 25,792 and 12,457 unigenes annotated in the UniProt, Pfam and eggNOG databases, respectively.

For the NR annotation, 40,796 unigenes had hits in the NR database (Table S2). The *E*-value distribution pattern showed that 46.83% of the top hits had high homology with an *E*-value < 1e^−50^ (Fig. S3A). For identification, more than 71% of the sequences had a similarity higher than 60%, and most of the annotated unigenes had identified distributions that ranged from 60 to 80% (Fig. S3B). On a species basis, the annotated sequences had identical fragments with genes from *Actinidia chinensis* var. chinensis (22.66%), *Vitis vinifera* (10.47%) and *Quercus suber* (3.85%) (Fig. S3C).

All of the unigenes were categorized according to the secondary classification of GO terms. A total of 29,824 unigenes were assigned to a GO term in three main GO classification categories: biological process, cellular component and molecular function (Fig. S4 and Table S2). The major classes of the biological process category were DNA metabolic process, biosynthetic process and cellular nitrogen compound metabolic process. The terms cellular component, nucleus and cytoplasm were dominant in the cellular component category. The main molecular function category terms were ion binding, molecular function and kinase activity.

KEGG pathway analysis revealed that 3,017 unigenes were assigned to 341 pathways (Tables S2 and S3). The major enrichments among metabolic pathways were carbon metabolism, biosynthesis of amino acids, ABC transporters, purine metabolism, two-component system and pyruvate metabolism. The metabolic pathways related to leaf color were porphyrin and chlorophyll metabolism (ko00860; 50 unigenes), photosynthesis (ko00195, 23 unigenes), carotenoid biosynthesis (ko00906, 11 unigenes), flavonoid biosynthesis (ko00941, 5 unigenes), and flavone and flavonol biosynthesis (ko00944, 3 unigenes).

### Differentially expressed genes (DEGs) and functional analysis

**Fig. 5:**
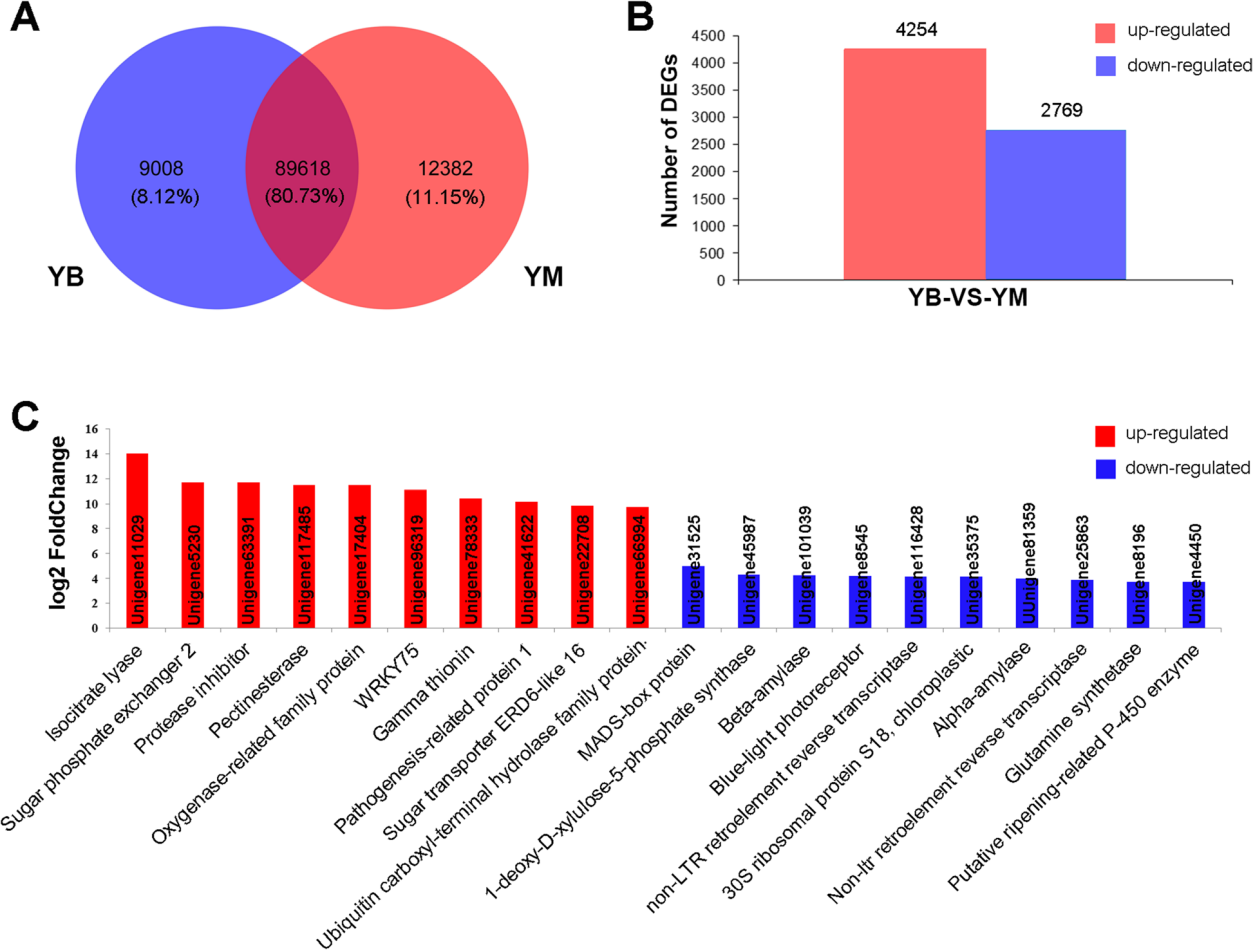
The number of genes and differentially expressed genes were detected in *H. macrophylla* var. *maculata* (**YB**) and full-green leaf mutant (**YM**). (**A**) The number of genes was detected in the YB and YM libraries. (**B**) Differentially expressed genes were detected between YB and YM. (**C**) Top 10 significantly up- and down-regulated genes whose sequences were annotated were detected between YB and YM

Fragments per kilobase per million (FPKM) was used to estimate the expression levels of genes. As a result, 98,626 and 102,000 unigenes were identified in the YB and YM libraries, respectively (Table S4). A total of 9,008 (8.12%) and 12,382 (11.15%) unigenes were expressed specifically in the leaves of YB and YM, respectively, and 89,618 (80.73%) unigenes were co-expressed in YB and YM (Fig. 5A). To identify DEGs between YB and YM, the expression of genes in YM was set as the control. A total of 7,023 genes showed at least a two-fold change in gene expression level, including 4,254 up-regulated and 2,769 down-regulated genes (Fig. 5B and Table S5). The top 10 significantly up- and down-regulated genes whose sequences were annotated were detected between YB and YM (Fig. 5C). These genes may be involved in leaf color formation.

The functions of the DEGs were categorized according to the secondary classification of GO terms. The results showed that 203, 192 and 454 DEGs were divided into biological process, cellular component and molecular function categories, respectively (Fig. S5 and Table S6). The most enriched terms of the biological process were cell wall organization, signal transduction and lipid metabolic process. Many DEGs in the cellular component category were associated with the membrane, plasmodesma and extracellular region. The dominant molecular function category terms were hydrolase activity, iron ion binding and heme binding.

KEGG pathway analysis was performed to categorize the DEGs. A total of 82 DEGs were enriched in 21 pathways in the KEGG database (Table S7). Plant hormone signal transduction was the most enriched pathway, followed by antigen processing and presentation. There were three DEGs in the photosynthesis pathway (Table S7). Compared to YM, the expression levels of photosystem, chlorophyll a-b binding protein, ATP synthase and cytochrome genes in YB were down-regulated (Table 2). This result further confirmed that the photosynthesis of YM was higher than YB.

**Table 2:** Differentially expressed genes involved in photosynthesis in the *H. macrophylla* transcriptome

Gene ID	log2(YB/YM)	Annotation
Unigene35224	-3.34	Photosystem II reaction center protein K
Unigene21595	-2.78	Photosystem II protein D1
Unigene83666	-2.42	Photosystem II CP47 reaction center protein
Unigene16923	-1.88	Photosystem II CP43 reaction center protein
Unigene71781	-1.81	Photosystem II D2 protein
Unigene27991	-1.32	Photosystem I P700 chlorophyll a apoprotein A1 family
Unigene105647	-1.31	Photosystem II type I chlorophyll a/b-binding protein
Unigene25859	-1.15	Photosystem II reaction center PsbP family protein
Unigene64241	-1.05	Photosystem I assembly protein ycf4
Unigene87772	-1.13	Chlorophyll a-b binding protein 2
Unigene30677	-1.94	ATP synthase subunit beta
Unigene78078	-2.08	Cytochrome f
Unigene116390	-1.33	Cytochrome b6
Unigene93247	-1.05	Cytochrome b6-f complex iron-sulfur subunit

### Analysis of genes related to chloroplast development and division and chlorophyll biosynthesis and degradation

Based on the *H. macrophylla* transcriptome data, the unigenes involved in chloroplast development and division and chlorophyll biosynthesis and degradation were identified (Table 3). Compared to YM, the expression levels of *DELLA*, *PPR*, *GLK* and *Thf1* (chloroplast development) and *FtsZ*, *MinD* and *AP2/ERF* (chloroplast division) in YB were down-regulated, and the expression levels of *ARFs* (chloroplast development) were up-regulated (Table 3). However, the unigenes involved in chlorophyll biosynthesis and degradation had no significant expression pattern changes between YB and YM (Table 3). These results indicated that the leaf color of YB may be caused by the expression pattern changes of chloroplast development and division genes.

**Table 3:** Unigenes involved in chloroplast development and division and chlorophyll biosynthesis and degradation in the *H. macrophylla* transcriptome

Function	Gene name	Gene ID	log2(YB/YM)	Annotation
chloroplast development	DELLA	Unigene73671	-1.42	DELLA protein GAI
		Unigene118449	-1.17	DELLA protein GAI-like
		Unigene119524	-3.21	GA repressor DELLA
	PPR	Unigene95411	-1.22	pentatricopeptide repeat-containing protein At1g08070
		Unigene644	-1.04	pentatricopeptide repeat-containing protein At2g15820
	GLK	Unigene20679	-2.17	transcription activator GLK1
	Thf1	Unigene88785	-1.28	thylakoid formation1
	ARF	Unigene48743	1.23	auxin response factor 17
		Unigene118329	1.94	Auxin response factor
		Unigene122697	1.15	Auxin response factor
		Unigene23042	1.02	Auxin response factor
		Unigene32938	1.21	Auxin response factor
		Unigene33148	1.79	Auxin response factor 17
		Unigene48743	1.23	Auxin response factor 17-like
		Unigene56446	1.25	Auxin response factor
		Unigene78971	1.21	Auxin response factor 1
chloroplast division	FtsZ	Unigene65459	-1.70	cell division protein FtsZ homolog 2–2, chloroplastic
		Unigene14090	-1.08	tubulin beta chain
		Unigene62710	-1.09	tubulin alpha chain
	MinD	Unigene3670	-1.31	Adenylyl-sulfate kinase
	AP2/ERF	Unigene42535	-1.43	AP2/ERF transcription factor
		Unigene17785	-1.30	AP2/ERF transcription factor
chlorophyll biosynthesis	HEMA	Unigene94259	0.30	Glutamyl-tRNA reductase
	GSA	Unigene21371	0.26	Glutamate-1-semialdehyde 2,1-aminomutase
	HEMB	Unigene80924	0.07	Delta-aminolevulinic acid dehydratase, chloroplastic
		Unigene54524	0.52	Delta-aminolevulinic acid dehydratase, chloroplastic
	HEMC	Unigene43517	0.62	Porphobilinogen deaminase
		Unigene80400	-0.54	Porphobilinogen deaminase
	HEMD	Unigene42585	-0.56	Uroporphyrinogen-III synthase
		Unigene106934	0.75	Uroporphyrinogen-III synthase
	HEME	Unigene3363	0.43	Uroporphyrinogen decarboxylase
		Unigene103027	0.30	Uroporphyrinogen decarboxylase 1, chloroplastic
		Unigene6415	0.41	Uroporphyrinogen decarboxylase
	HEMG	Unigene34893	0.19	Protoporphyrinogen oxidase
		Unigene106486	0.63	Protoporphyrinogen oxidase
	CHLH	Unigene1894	0.81	Magnesium-chelatase subunit ChlH like
	CHLI	Unigene48515	0.55	Magnesium-chelatase subunit ChlI, chloroplastic-like
	CHLM	Unigene22407	-0.59	Mg-protoporphyrin IX methyltransferase
	CHLG	Unigene25797	0.07	Chlorophyll synthase
	CAO	Unigene110336	-0.93	Chlorophyllide an oxygenase
chlorophyll degradation	NYC1	Unigene105397	0.03	Putative chlorophyll(Ide) b reductase NYC1
	NOL	Unigene114249	0.18	Chlorophyll b reductase NOL protein
	CLH1	Unigene91850	-0.49	chlorophyllase-1
		Unigene28136	-0.81	chlorophyllase-2
	PPH	Unigene14410	0.70	Pheophytinase
		Unigene43673	-0.03	pheophytinase
		Unigene47431	-0.55	pheophytinase
	PAO	Unigene110336	-0.93	Pheophorbide an oxygenase
		Unigene1999	-0.69	Pheophorbide an oxygenase
		Unigene74026	-0.10	Pheophorbide an oxygenase
		Unigene98774	-0.66	Pheophorbide an oxygenase
	RCCR	Unigene7212	-0.01	Red chlorophyll catabolite reductase

### Validation of DEGs using qRT–PCR

**Fig. 6:**
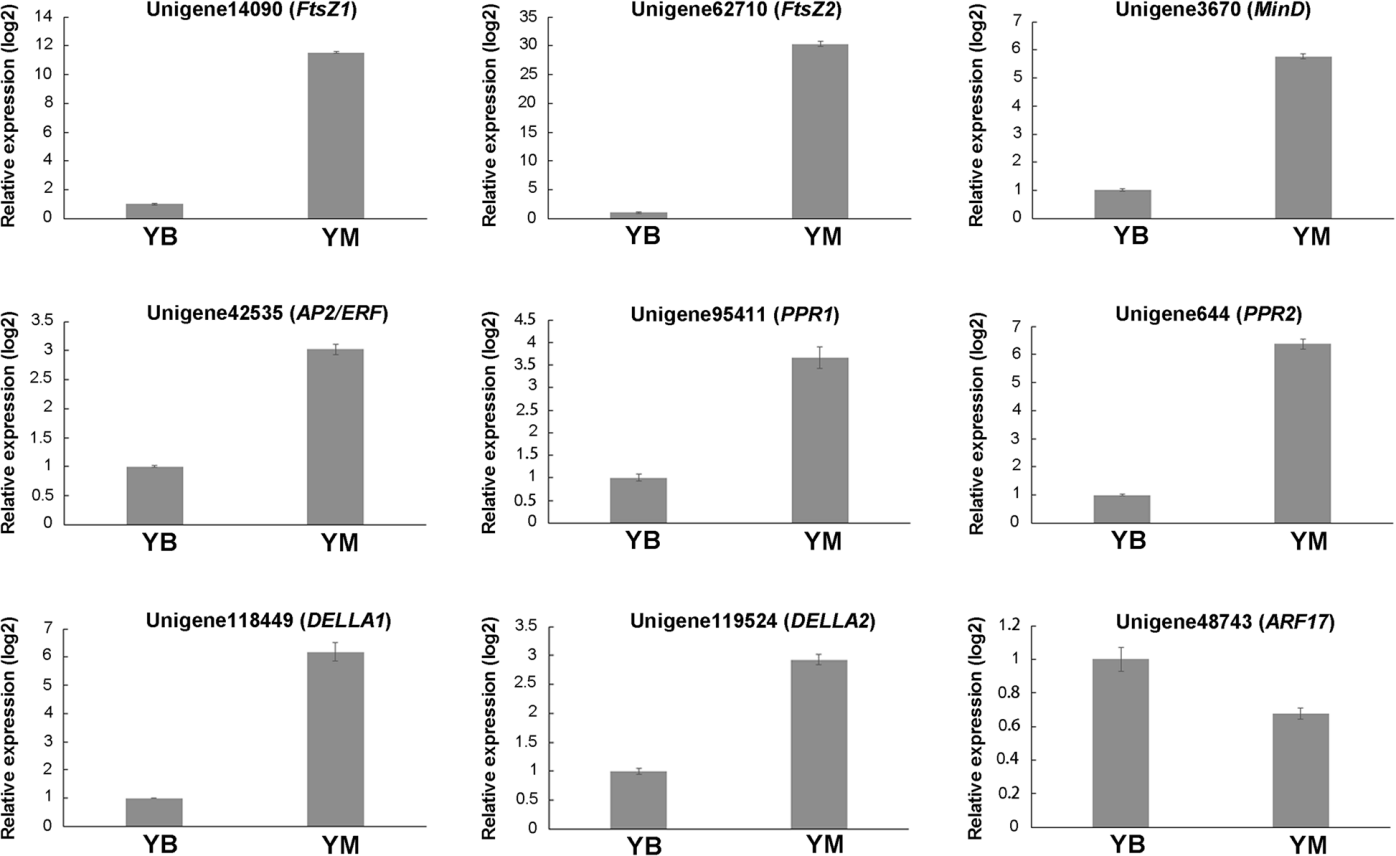
Verification of genes involved in chloroplast development and division using qRT–PCR between YB and YM

To verify the reliability of the transcriptome data, the expression patterns of the genes that were significantly expressed in the samples were verified using qRT–PCR (Fig. 6). The expression trends were generally consistent with the transcript abundances estimated from the RNA-Seq data, but the selected genes showed different fold-change values (Fig. 6 and Table 3). These results confirmed the reliability of the transcriptome data.

## Discussion

Chlorophyll and carotenoids are the major pigments in green leaves. Previous studies demonstrated that leaf color mutants commonly contained less chlorophyll and carotenoids [13, 15, 18]. For example, the contents of pigments, including chlorophyll *a*, chlorophyll *b*, total chlorophyll and carotene, were decreased in the durum wheat mutant [28]. The present study obtained a mutant with green leaves (YM) from the YB plant. Consistent with previous reports, the pigment contents were lower in YB than YM (Fig. 3). Photosynthesis is a complex process that is easily affected by changes in pigment contents [29]. Physiological experiments showed that photosynthesis was restricted in YB (Fig. 2). Therefore, there may be fewer light-harvesting protein complexes in YB than YM, which was verified by the results that showed that the down-regulated DEGs were primarily enriched in photosynthesis (Table 2). These results revealed that the expression changes of genes involved in photosynthesis played an important role in the formation of the capacity of plant photosynthesis. Previous research reported that the expression change of genes related to photosynthesis in *Brassica campestris* mutants led to abnormal chloroplast development and reduced pigment content [30]. Therefore, the decreased capacity of photosynthesis in YB was likely due to the reduction of pigment contents and the low expression level of photosystem, chlorophyll a-b binding protein, ATP synthase and cytochrome genes.

Chloroplast development of higher plants requires the coordination of nuclear genes and chloroplast genes [31]. The DELLA proteins that accumulate in the nucleus are key suppressors of GA responses via inhibition of GA-regulated gene expression [32]. DELLA proteins act negatively in GA responses by interacting with diverse regulators or transcription factors [33, 34]. GA levels are reduced under light, which stimulates DELLA accumulation and abolishes negative control by DELLA targets, including PIFs that are suppressors of chloroplast development [35, 36]. Analysis of *della* mutants revealed the complicated regulation of chloroplast development, and it was reported that GA prevented photomorphogenesis in the dark [37]. As nuclear factors, PPR proteins are involved in the expression of chloroplast genes in many post-transcriptional processes [38, 39]. *AtECB2* (a *PPR* gene) regulated the editing of the *accD* and *ndhF* genes in *Arabidopsis* early chloroplast biogenesis, and the *ecb2* mutant showed a lack of thylakoid membranes with a delayed greening phenotype [40]. *GhYGL1d* (a *PPR* gene) regulated the development of thylakoids in cotton by editing the *accD* and *ndhF* genes, therefore, *GhYGL1d*-silenced cotton exhibited significant abnormalities in thylakoid structures compared to wild-type cotton [41]. Previous studies suggested that *GLK* genes were involved in regulating chloroplast development in plant species [22, 42, 43]. Members of the *GLK* gene family are sensitive to chloroplast retrograde signaling, and they control downstream genes for plastid retrograde signaling [44]. Compared to the wild type *A. andraeanum*, *GLK* was downregulated in the *rubescent* mutant [13]. The *A. thaliana thylakoid formation1* (*Thf1*) gene controls vesicles maturation into thylakoid stacks and ultimately for leaf development, and deletion of *AtThf1* leads to deficient thylakoid formation and variegated leaves [45]. Consistent with these studies, the expression levels of *HmDELLA*, *HmPPR*, *HmGLK* and *HmThf1* in YB were lower than YM in the present study (Fig. 6 and Table 3), which indicates that these genes are related to chloroplast development.

Auxin is involved in photomorphogenesis, as proposed by the phenotype of dark-induced hypocotyl elongation in auxin-response mutants [46]. Auxin response factors (ARFs), which bind to promoters of auxin-responsive genes to regulate transcription, mediate numerous auxin responses. ARF function is suppressed by auxin/indole-3-acetic acid inducible (Aux/IAA) proteins. Auxin negatively regulates root greening via IAA14/SLR, ARF7 and ARF19 [47]. Overexpression of *CYTOKININ-RESPONSIVE GATA FACTOR 1* (*CGA1*) and *GATA, NITRATE-INDUCIBLE, CARBON-METABOLISMINVOLVED* (*GNC*) promote the differentiation of etioplasts to chloroplasts in the light [48]. ARF2 binds to the promoters of *CGA1* and *GNC*, suppresses their expression, and *arf2* mutation promotes root greening [49]. The expression levels of *HmARFs* in YB were higher than YM (Fig. 6 and Table 3), which is consistent with previous reports and suggests the involvement of *HmARFs* in chloroplast development.

Inhibiting the expression of *FtsZ* genes in transgenic *Arabidopsis* plants significantly reduced the number of chloroplasts in mature leaves, which indicates that *FtsZ* genes are essential for the division of plant chloroplasts [50, 51]. The proteins AtFtsZ, AtMinD and AtMinE act in concert during chloroplast division [52]. The expression patterns of *FtsZ* and *MinD* in YB were similar to the *A. andraeanum rubescent* mutant [13], which indicates that *HmFtsZ* and *HmMinD* are related to chloroplast division.

Cytokinin response factor 2 (CRF2) belongs to the APETLA2/ETHYLENE RESPONSE FACTOR (AP2/ERF) transcription factor family [53]. Overexpression of *CRF2* resulted in an increased level of *Plastid Division 2* (*PDV2*) and promoted chloroplast division [54]. The expression levels of *HmAP2/ERFs* (Unigene42535 and Unigene17785) in YB were lower than YM in the present study (Fig. 6 and Table 3), which indicates that *HmAP2/ERF* is involved in chloroplast division.

## Conclusions

In conclusion, the photosynthesis and pigment contents were reduced in YB compared to YM, and the expression levels of many genes related to chloroplast development and division were changed. These results suggest that the change in gene expression patterns involved in chloroplast development and division are responsible for the abnormal ultrastructure of chloroplasts, which results in the silvery white leaf edges in YB. Our results provide a basis for further research on leaf color mutation.

## Methods

### Plant materials

YM was a bud mutation derived from YB (Fig. 1A), which varied only in leaf color (Fig. 1). The YB and YM were maintained in the Preservation Centre of the Hydrangea Germplasm Resources, Jiangsu Academy of Agricultural Sciences, Nanjing, China (latitude: 32°05′N, longitude: 118°08′E; 68 m above sea level) (Fig. 1B, E). The materials were propagated by cuttings. The plants were grown in a greenhouse (25 °C during the day and 15 °C at night; relative humidity of 60–70%; under natural light).

### SRAP analysis

DNA was extracted from the fourth leaf of three individual YB and YM plants using a modified CTAB method [55]. The DNA was used for SRAP profiling as described by Li and Quiros [56]. A total of 136 SRAP primer pairs were used, including 24 forward and 20 reverse primers (Table S8). The pairs were M1 combined with E1 (abbreviated “M1/E1”), M1/E16, M1/E19, M2/E1, M2/E2, M2/E6, M2/E12, M2/E15, M2/E18, M3/E1, M3/E2, M3/E3, M3/E5, M3/E11, M3/E17, M4/E3, M4/E4, M4/E6, M4/E8, M4/E11, M5/E1, M5/E3, M5/E5, M5/E7, M5/E8, M6/E1, M6/E2, M6/E6, M6/E11, M6/E14, M6/E18, M6/E19, M7/E2, M7/E4, M7/E6, M7/E7, M7/E15, M7/E17, M7/E20, M8/E3, M8/E5, M8/E8, M8/E12, M8/E15, M8/E16, M8/E18, M9/E9, M9/E15, M9/E18, M10/E1, M10/E2, M10/E4, M10/E10, M11/E2, M11/E5, M11/E8, M11/E10, M11/E11, M11/E16, M12/E2, M12/E7, M12/E10, M12/E12, M12/E13, M12/E15, M12/E17, M13/E3, M13/E6, M13/E8, M13/E13, M13/E16, M14/E2, M14/E5, M14/E7, M14/E10, M14/E11, M14/E14, M14/E16, M15/E1, M15/E10, M15/E15, M15/E17, M15/E20, M16/E2, M16/E10, M16/E11, M16/E14, M16/E15, M16/E16, M16/E19, M17/E1, M17/E5, M17/E7, M17/E15, M17/E19, M18/E6, M18/E8, M18/E10, M18/E14, M18/E18, M19/E5, M19/E7, M19/E12, M19/E16, M19/E18, M19/E19, M20/E2, M20/E4, M20/E8, M20/E13, M21/E1, M21/E2, M21/E4, M21/E6, M21/E14, M21/E17, M21/E19, M22/E1, M22/E2, M22/E5, M22/E6, M22/E11, M22/E15, M22/E20, M23/E3, M23/E6, M23/E10, M23/E12, M23/E16, M23/E18, M24/E2, M24/E4, M24/E8, M24/E11, M24/E15 and M24/E16. Each 25 μl reaction mix was comprised of 2.5 μl of 10 × PCR buffer, 0.2 mM dNTPs, 1.5 mM MgCl_2_, 15 ng genomic DNA and 2 U of Taq polymerase (Takara, Japan). The reactions were first denatured (94 °C/5 min), followed by 5 cycles of 94 °C/1 min, 35 °C/1 min and 72 °C/2 min, followed by 35 cycles of 94 °C/1 min, 50 °C/1 min and 72 °C/2 min, with a final extension step of 72 °C/10 min. The SRAP amplicons were electrophoresed with 6% denaturing polyacrylamide gels and visualized via silver staining. Fragments in the size range of 100–500 bp were scored.

### Photosynthetic parameters

Photosynthetic parameters consisting of Pn (µmol CO_2_ m^−2^ s^−1^), Gs (mol H_2_O m^−2^ s^−1^), Ci (µmol CO_2_ mol^−1^) and Tr (mol H_2_O m^−2^ s^−1^) were determined using an LI-6400 portable photosynthesis system (LI-COR, Lincoln, NE, USA). These data were recorded between 9:00 and 11:00 am using the first most fully expanded leaves from the apex of the shoot. The air cuvette temperature, CO_2_ concentration and irradiance were maintained at 30 °C, 420 µmol CO_2_ mol^−1^ and 1000 µmol m^−2^ s^−1^, respectively [57]. Ten representative plants of YB and YM were selected randomly and determined.

### Chlorophyll and carotenoid content

Each leaf was cut into edged leaves (EL) and central leaves (CL) (Fig. 1C and 1F). Chlorophyll a, chlorophyll b and total carotenoid contents were measured using the methods of Zhang et al. [58]. Approximately 200 mg (fresh weight) of the fourth leaf from the stem tip was incubated in 10 mL 95% ethanol for 48 h in the dark. The absorbance of the supernatant was analyzed using spectrophotometry (UH5300, HITACHI, Tokyo, Japan) at 665, 649 and 470 nm. Total Chl (Chl a + Chl b) was also estimated. Three biological replicates were performed for each sample. Data were compared using analysis of variance (Duncan’s multiple range tests at *p *= 0.05) using SPSS v17.0 software (SPSS Inc.,Chicago, IL, USA).

### Chloroplast ultrastructure

To observe the chloroplast ultrastructure of mesophyll cells, edge leaves of YB and YM 0.5 cm × 0.5 cm in size were immediately fixed in fresh 2.5% (v/v) glutaraldehyde (0.1 mol L^−1^ phosphate buffer, pH 7.2) for at least 48 h (Fig. 1C, F). The samples were immersed in 1% (v/v) osmium acid for post-fixation, embedded in resin and imaged using a transmission electron microscope (H7650, HITACHI, Tokyo, Japan).

### RNA extraction and RNA-Seq analysis

The edged leaves of the fourth leaf from three individual YB and YM plants were harvested and snap frozen in liquid nitrogen. Three biological replicates were used for RNA-Seq analysis. Total RNA was extracted using RNAiso reagent (Takara, Japan) according to the manufacturer’s instructions. The quality and integrity of the total RNA were verified using a 2100 Bioanalyzer RNA Nano chip device (Agilent, Santa Clara, CA, USA). The concentration was measured using an ND-430 1000 spectrophotometer (NanoDrop, Wilmington, DE). The RNA was stored at -80 °C for subsequent use.

The mRNA of each library was sequenced on an Illumina NovaSeq 6000 platform located at Wuhan Benagen Tech Solution Co. Ltd. (Wuhan, China; http://www.benagen.com). To obtain high-quality clean reads, adapters, reads containing more than 5% poly-N and low-quality reads were removed from the raw data. The Q20, Q30 and GC contents of the clean data were calculated. De novo assembly was performed using Trinity (http://trinityrnaseq.github.io) [59]. The remaining clean reads were also spliced into unigenes by the same software. The NR (ftp://ftp.ncbi.nlm.nih.gov/blast/db), eggNOG (http://eggnogdb.embl.de/#/app/home), UniProt (http://www.uniprot.org/) [60], Pfam (v30.0) (http://pfam.xfam.org/) [61] and KEGG (v79.1) (http://www.genome.jp/kegg) [62] databases were used for blast search and annotation. All unigenes were first searched in the NR database with an *E*-value ≤ 10^–5^. Blast (v2.2.28 +) (http://blast.ncbi.nlm.nih.gov/Blast.cgi) [63] and DIAMOND (v0.7.11) (https://github.com/bbuchfink/diamond) [64] were used for BLAST search and annotation. HMMER (v3.1) (http://hmmer.org/) was used for domain annotation [65]. ClusterProfiler (v3.6.0) (http://www.bioconductor.org/packages/release/bioc/html/clusterProfiler.html) was used to obtain the GO and KEGG pathway annotations [66].

After obtaining the number of read counts of the samples, FPKM was used to estimate the expression levels of genes and compare differences in gene expression between YB and YM. DEGs were identified using an algorithm developed by Audic and Claverie [67]. The criteria applied to the thresholds for significant differences in gene expression were *P*-values ≤ 0.05, a false discovery rate (FDR) ≤ 0.05 and |log_2_Ratio|≥ 1.0.

### qRT–PCR validation of DEGs

Total RNA was extracted from the edged leaf of the fourth leaf from three individual YB and YM plants using RNAiso reagent (Takara, Japan) according to the manufacturer’s recommendations. Primers were designed in Primer 5.0 software using the sequences from the transcriptome (Table S9). The *H. macrophylla 18S rRNA* gene was used as the reference [68]. The qRT–PCR mixtures were prepared following the instructions of the TB Green^®^*Premin Ex Taq*™ reagent kit (Takara, Japan). qRT–PCR was performed on a 7500 Real-Time PCR System (Applied Biosystems, CA, USA). The PCR cycles were first denatured (95 °C/30 s), followed by 40 cycles of 95 °C/5 s, 60 °C/34 s, and finally 1 cycle of 95 °C/15 s, 60 °C/60 s, 95 °C/15 s. Three biological replicates and three technical replicates were used for qRT–PCR analysis. Relative expression levels were calculated using the 2^−**△△**CT^ method.

## Supplementary Information

**Additional file 1: Supplementary fig 1.** SRAP profiling of *H. macrophylla* var. *maculate* (YB) and full-green leaf mutant (YM). M: Marker, 1: YB, 2: YM. M23+E3, M23+E6, M23+E10, M23+E12, M23+E16, M23+E18, M24+E2, M24+E4, M24+E8, M24+E11, and M24+E15 represented primer pairs. 

**Additional file 2: Supplementary fig 2.** The distribution of unigenes length.  

**Additional file 3: Supplementary fig 3.** The distribution of E-value (A), identify (B) and species (C) of *H. macrophylla* unigenes against non-redundant database.  

**Additional file 4: Supplementary fig 4.** Gene ontology functional classification of *H. macrophylla* unigenes. Unigenes were annotated in three categories: biological process (red), cellular component (green) and molecular function (blue)  

**Additional file 5: Supplementary fig 5.** Gene ontology functional classification of differentially expressed genes. DEGs were annotated in three categories: biological process (red), cellular component (green), and molecular function (blue). 

**Additional file 6: Supplementary table S1.** Reading mapping summary of *H. macrophylla*. 

**Additional file 7: Supplementary table S2.** The annotated genes of *H. macrophylla* that may be functionally classified in each corresponding database.

**Additional file 8: Supplementary table S3.** Pathway classification of all the unigenes in *H. macrophylla*.

**Additional file 9: Supplementary table S4.** The expression of all unigenes in *H. macrophylla*.

**Additional file 10: Supplementary table S5.** The expression of DEGs in YB-VS-YM.

**Additional file 11: Supplementary table S6.** The GO significant enrichment analysis of DEGs in YB-VS-YM.

**Additional file 12: Supplementary table S7.** Pathway classification of the DEGs in *H. macrophylla*.

**Additional file 13: Supplementary table S8.** Primer sequences used for amplification in SRAP analysis.

**Additional file 14: Supplementary table S9.** Primer sequences for qRT–PCR.

## Data Availability

The raw data of transcriptome sequencing reads of *H. macrophylla* have been deposited in NCBI under BioProject accession number PRJNA778011 and BioSample accession numbers SAMN22898762 and SAMN22898763. The file of unigene sequences is available publicly in figshare (https://doi.org/10.6084/m9.figshare.19625256.v1).

## Abbreviations

HEMA: Glutamyl-tRNA reductase
CAO: Chlorophyllide an oxygenase
CHLI: Magnesium-chelatase I subunit
PPR: Pentatricopeptide repeat
GLK: Golden 2-like
SRAP: Sequence-related amplified polymorphism
FPKM: Fragments per kilobase per million
DEGs: Differentially expressed genes
qRT–PCR: Quantitative real-time PCR
ARF: Auxin response factor
