# Supplementary figures and images for: Comparative physiology and transcriptome analysis reveals that chloroplast development influences silver-white leaf color formation in Hydrangea macrophylla var. maculata
Source: BMC Plant Biol. 2022 Jul 16;22:345. doi: 10.1186/s12870-022-03727-1 (PMC9287875; doi:10.1186/s12870-022-03727-1)

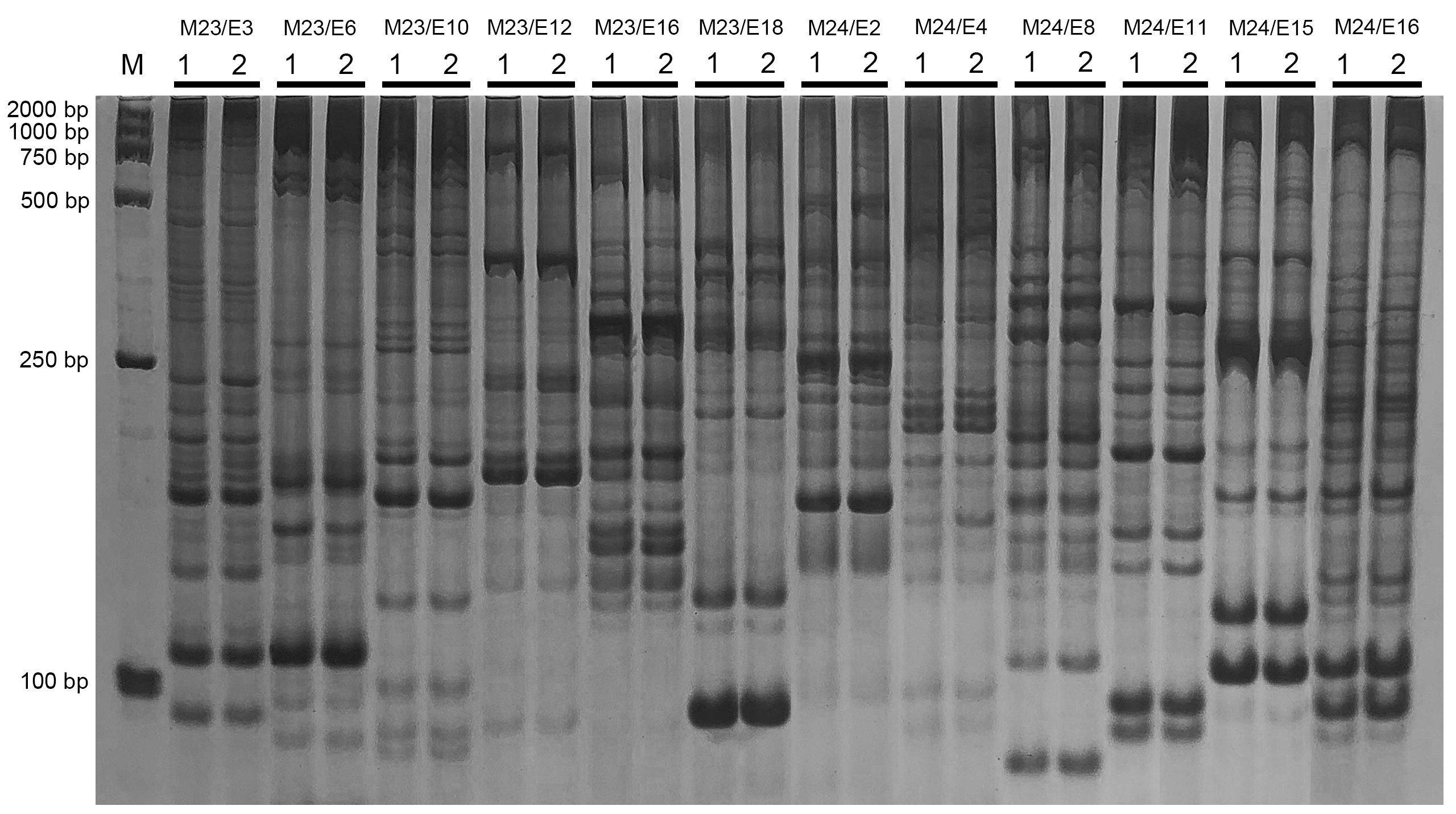

Supplement: Supplementary file 1 — Additional file 1: Supplementary fig 1. SRAP profiling of H. macrophylla var. maculate (YB) and full-green leaf mutant (YM). M: Marker, 1: YB, 2: YM. M23+E3, M23+E6, M23+E10, M23+E12, M23+E16, M23+E18, M24+E2, M24+E4, M24+E8, M24+E11, and M24+E15 represented primer pairs. [file 12870_2022_3727_MOESM1_ESM.tif]

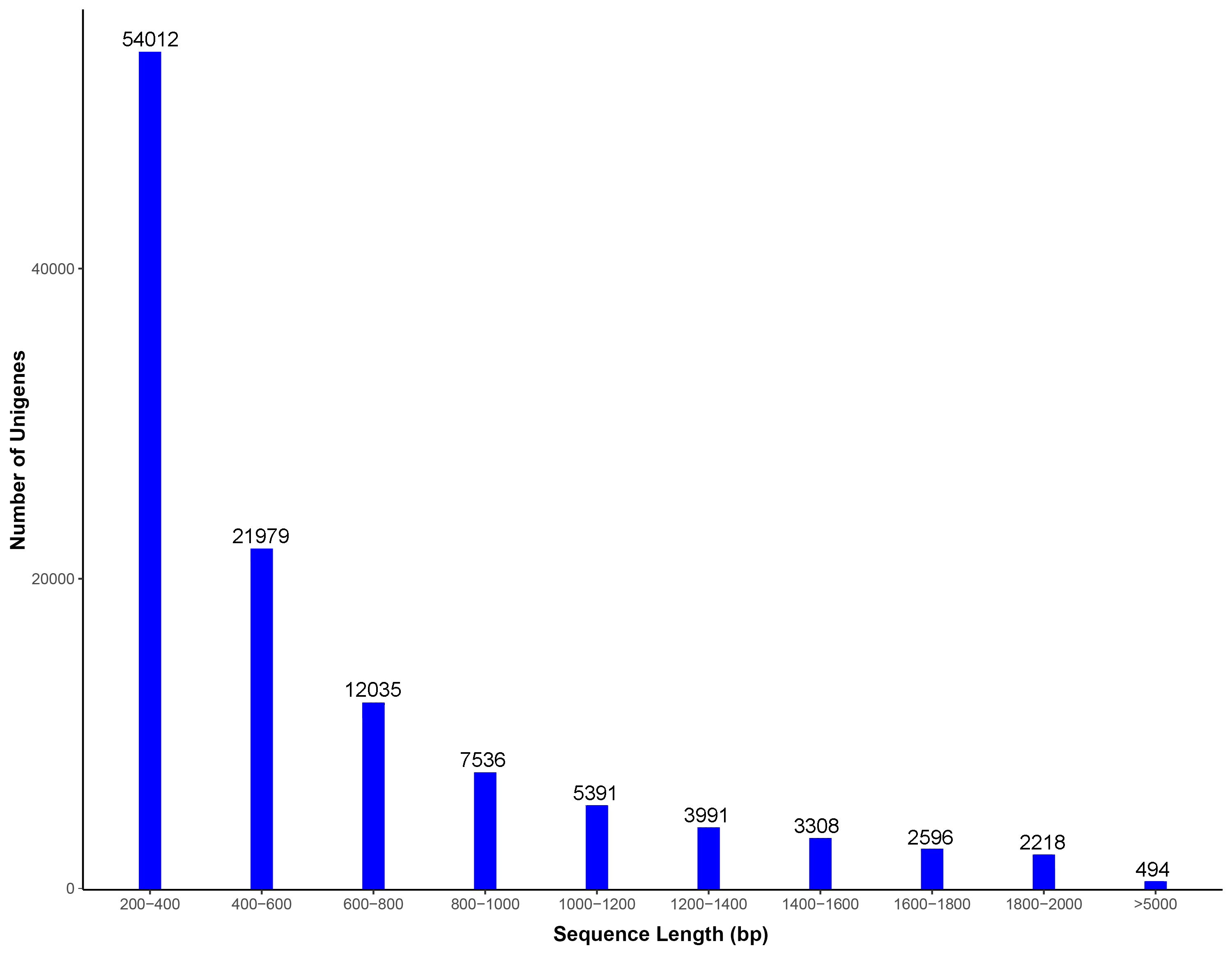

Supplement: Supplementary file 2 — Additional file 2: Supplementary fig 2. The distribution of unigenes length. [file 12870_2022_3727_MOESM2_ESM.tif]

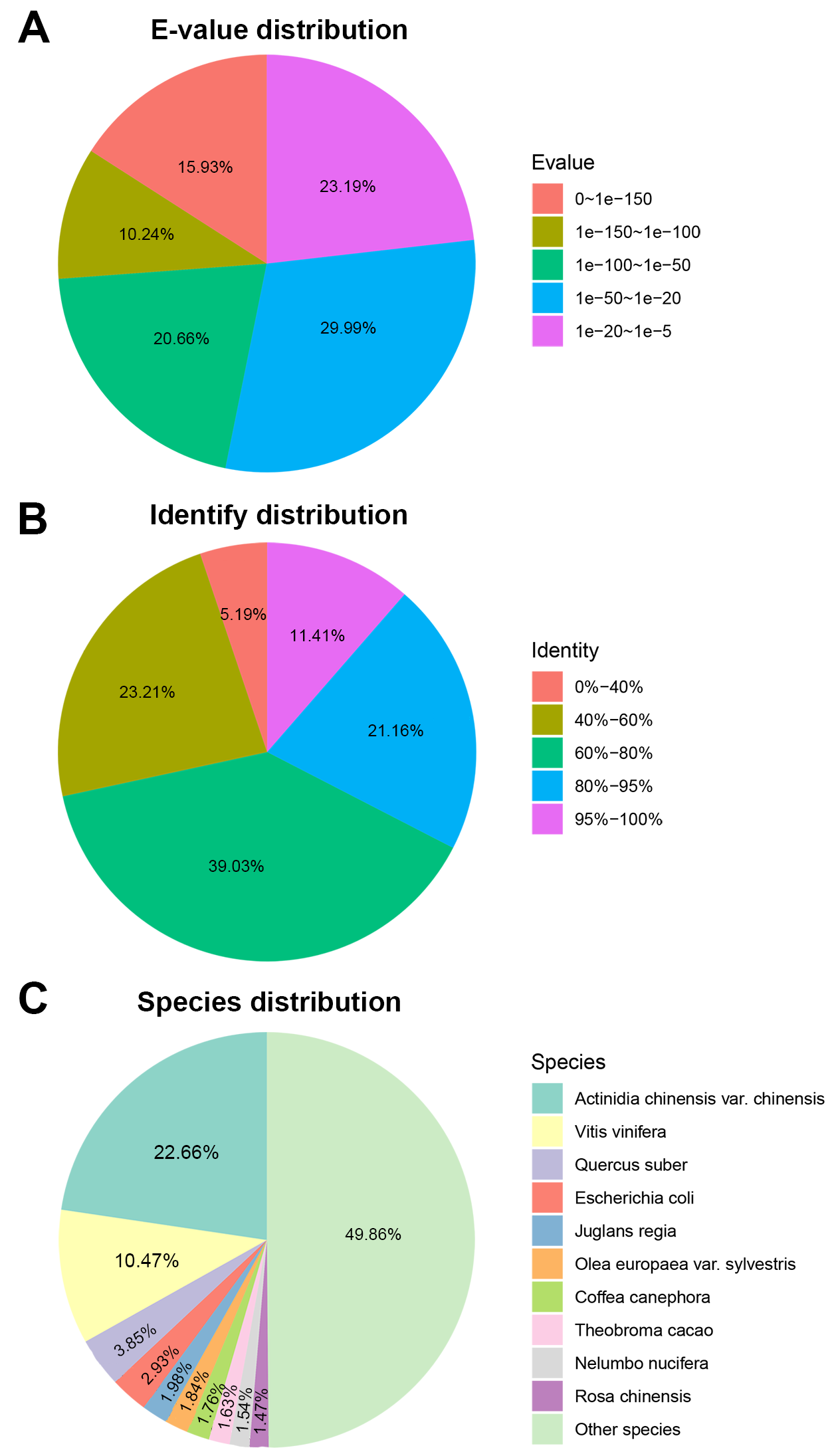

Supplement: Supplementary file 3 — Additional file 3: Supplementary fig 3. The distribution of E-value (A), identify (B) and species (C) of H. macrophylla unigenes against non-redundant database. [file 12870_2022_3727_MOESM3_ESM.tif]

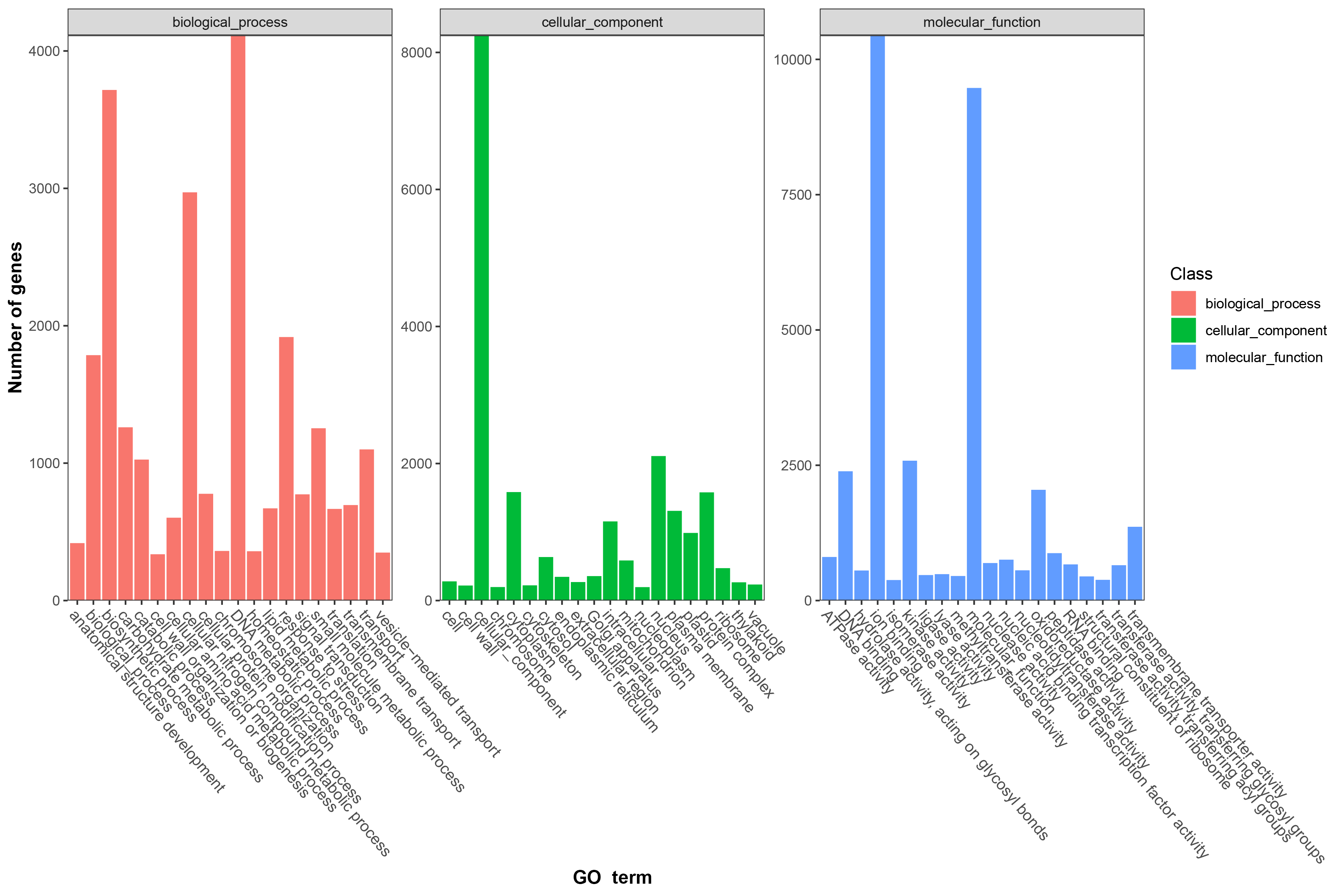

Supplement: Supplementary file 4 — Additional file 4: Supplementary fig 4. Gene ontology functional classification of H. macrophylla unigenes. Unigenes were annotated in three categories: biological process (red), cellular component (green) and molecular function (blue) [file 12870_2022_3727_MOESM4_ESM.tif]

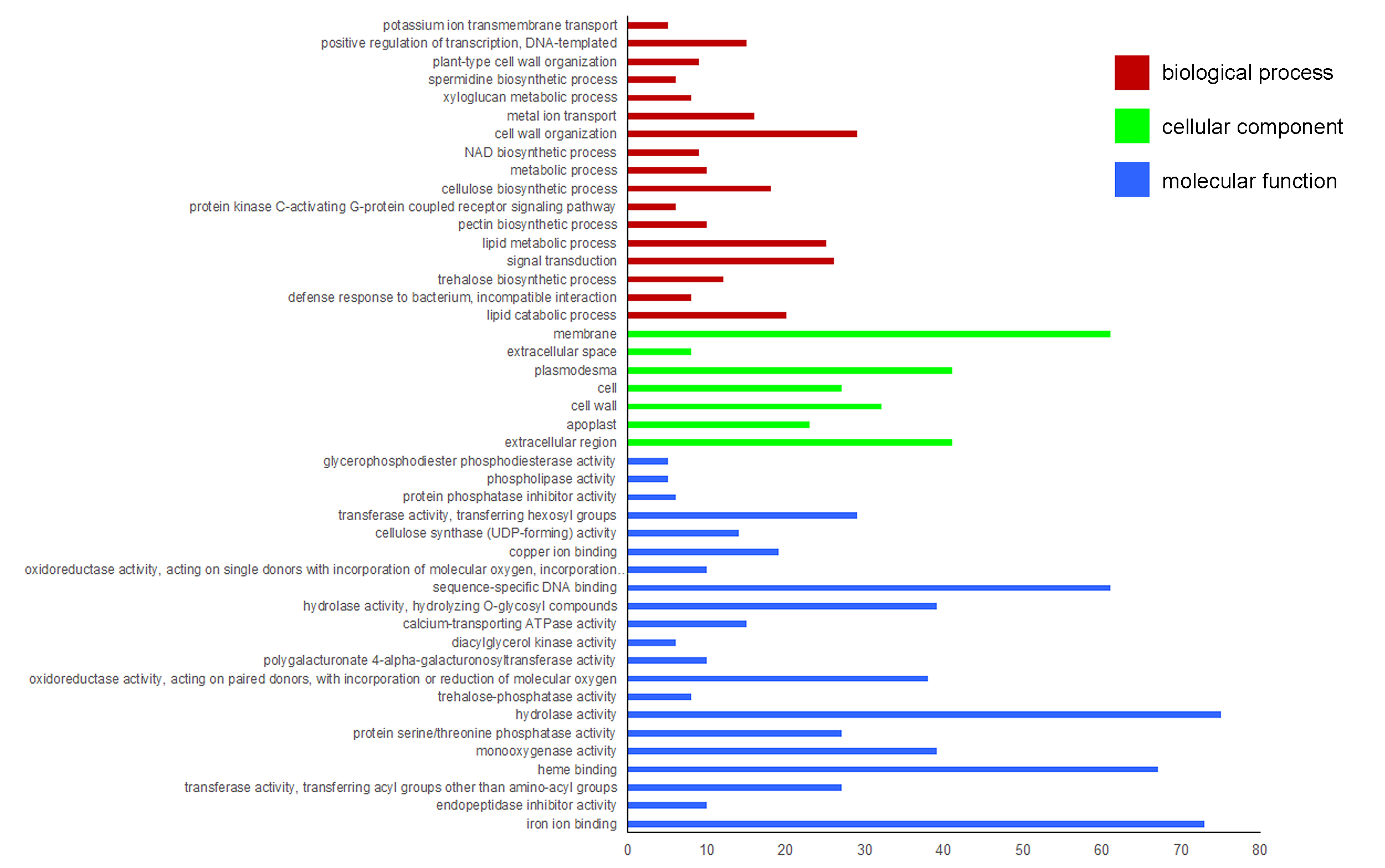

Supplement: Supplementary file 5 — Additional file 5: Supplementary fig 5. Gene ontology functional classification of differentially expressed genes. DEGs were annotated in three categories: biological process (red), cellular component (green), and molecular function (blue). [file 12870_2022_3727_MOESM5_ESM.tif]
