# Supplementary material for: Comparative physiology and transcriptome analysis reveals that chloroplast development influences silver-white leaf color formation in Hydrangea macrophylla var. maculata
Source: BMC Plant Biol. 2022 Jul 16;22:345. doi: 10.1186/s12870-022-03727-1 (PMC9287875; doi:10.1186/s12870-022-03727-1)
Supplement: Supplementary file 6 — Additional file 6: Supplementary table S1. Reading mapping summary of H. macrophylla. [file 12870_2022_3727_MOESM6_ESM.docx]

Table S1 Reading mapping summary of *H. macrophylla*.

| Sample | Total raw reads | Total raw data (bp) | Total clean reads | Total clean data (bp) | Clean reads ratio (%) | Clean data ratio (%) |
| --- | --- | --- | --- | --- | --- | --- |
| YB-1 | 47,956,778 | 7,193,516,700 | 47,950,946 | 7,192,641,900 | 99.98 | 99.98 |
| YB-2 | 54,905,974 | 8,235,896,100 | 54,898,964 | 8,234,844,600 | 99.98 | 99.98 |
| YB-3 | 48,389,498 | 7,258,424,700 | 48,384,546 | 7,257,681,900 | 99.99 | 99.98 |
| YM-1 | 50,247,890 | 7,537,183,500 | 50,240,646 | 7,536,096,900 | 99.98 | 99.98 |
| YM-2 | 50,621,570 | 7,593,235,500 | 50,613,226 | 7,591,983,900 | 99.98 | 99.98 |
| YM-3 | 64,679,848 | 9,701,977,200 | 64,664,862 | 9,699,729,300 | 99.97 | 99.97 |
