# Supplementary material for: Comparative physiology and transcriptome analysis reveals that chloroplast development influences silver-white leaf color formation in Hydrangea macrophylla var. maculata
Source: BMC Plant Biol. 2022 Jul 16;22:345. doi: 10.1186/s12870-022-03727-1 (PMC9287875; doi:10.1186/s12870-022-03727-1)
Supplement: Supplementary file 7 — Additional file 7: Supplementary table S2. The annotated genes of H. macrophylla that may be functionally classified in each corresponding database. [file 12870_2022_3727_MOESM7_ESM.docx]

Table S2 The annotated genes of *H. macrophylla* that may be functionally classified in each corresponding database.

| Database | Number | Percentage |
| --- | --- | --- |
| Annotation | 42,572 | 34.58% |
| Uniprot | 40,235 | 32.68% |
| Pfam | 25,792 | 20.95% |
| GO | 29,824 | 24.22% |
| KEGG | 3,017 | 2.45% |
| eggNOG | 12,457 | 10.12% |
| Nr | 40,796 | 33.14% |
| All | 123,122 | 100% |
