# Supplementary material for: Comparative physiology and transcriptome analysis reveals that chloroplast development influences silver-white leaf color formation in Hydrangea macrophylla var. maculata
Source: BMC Plant Biol. 2022 Jul 16;22:345. doi: 10.1186/s12870-022-03727-1 (PMC9287875; doi:10.1186/s12870-022-03727-1)
Supplement: Supplementary file 13 — Additional file 13: Supplementary table S8. Primer sequences used for amplification in SRAP analysis. [file 12870_2022_3727_MOESM13_ESM.docx]

Table S8 Primer sequences used for amplification in SRAP analysis.

| Primers | Forward primer | Primers | Reverse primer |
| --- | --- | --- | --- |
| M1 | TGAGTCCAAACCGGATA | E1 | GACTGCGTACGAATTAAT |
| M2 | TGAGTCCAAACCGGAGC | E2 | GACTGCGTACGAATTTGC |
| M3 | TGAGTCCAAACCGGAAT | E3 | GACTGCGTACGAATTGAC |
| M4 | TGAGTCCAAACCGGACC | E4 | GACTGCGTACGAATTTGA |
| M5 | TGAGTCCAAACCGGAAG | E5 | GACTGCGTACGAATTAAC |
| M6 | TGAGTCCAAACCGGTAG | E6 | GACTGCGTACGAATTGCA |
| M7 | TGAGTCCAAACCGGTTG | E7 | GACTGCGTACGAATTATG |
| M8 | TGAGTCCAAACCGGTGT | E8 | GACTGCGTACGAATTAGC |
| M9 | TGAGTCCAAACCGGTCA | E9 | GACTGCGTACGAATTACG |
| M10 | TGAGTCCAAACCGGATG | E10 | GACTGCGTACGAATTTAG |
| M11 | TGAGTCCAAACCGGACA | E11 | GACTGCGTACGAATTTCG |
| M12 | TGAGTCCAAACCGGGAT | E12 | GACTGCGTACGAATTGTC |
| M13 | TGAGTCCAAACCGGTAA | E13 | GACTGCGTACGAATTGGT |
| M14 | TGAGTCCAAACCGGGCT | E14 | GACTGCGTACGAATTCAG |
| M15 | TGAGTCCAAACCGGTGC | E15 | GACTGCGTACGAATTCTG |
| M16 | TGAGTCCAAACCGGAGG | E16 | GACTGCGTACGAATTCGG |
| M17 | AGCGAGCAAGCCGGTGG | E17 | GACTGCGTACGAATTCCA |
| M18 | GAGCGTCGAACCGGATG | E18 | GACTGCGTACGAATTGAT |
| M19 | CAAATGTGAACCGGATA | E19 | TGTGGTCCGCAAATTTAG |
| M20 | GAGTATCAACCCGGATT | E20 | AGGCGGTTGTCAATTGAC |
| M21 | GTACATAGAACCGGAGT |  |  |
| M22 | TACGACGAATCCGGACT |  |  |
| M23 | CACAGTCATGCCGGAAT |  |  |
| M24 | GACCAGTAAACCGGATG |  |  |
