# Supplementary material for: Comparative physiology and transcriptome analysis reveals that chloroplast development influences silver-white leaf color formation in Hydrangea macrophylla var. maculata
Source: BMC Plant Biol. 2022 Jul 16;22:345. doi: 10.1186/s12870-022-03727-1 (PMC9287875; doi:10.1186/s12870-022-03727-1)
Supplement: Supplementary file 14 — Additional file 14: Supplementary table S9. Primer sequences for qRT–PCR. [file 12870_2022_3727_MOESM14_ESM.docx]

| Gene ID | Gene name | Forward primer | Reverse primer |
| --- | --- | --- | --- |
| Unigene14090 | FtsZ1 | TTCCCTGGTCAACTCAACTCTG | GTCAGAGGAGCGAAACCCA |
| Unigene62710 | FtsZ2 | AGTGCGGCATCAACTACCAG | GAGAACACCTCTGCCACGCT |
| Unigene3670 | MinD | GCCAAATGATTCCAATGGTAAG | TGCTGAAGTATCTCCTGCCTGT |
| Unigene42535 | AP2/ERF | TGTTTTGTGACTCGGTTGATGG | TCCAAAAGTGCCCGAAATG |
| Unigene95411 | PPR1 | CGAAAATGGTGGAACCTACAAG | CTTTGGTCCTTTTGATGTTTCC |
| Unigene644 | PPR2 | TCTGGAAACTGATTGAACCCC | CAGAATCATACCCACTAACAAAACC |
| Unigene118449 | DELLA1 | TGGTGGGGATGGTTACAGAGT | AGTTGCCAAGCCGAGGTG |
| Unigene119524 | DELLA2 | GGGCTCCGTCTCGCTGAA | GTTGTGGTTCGCCTCTTGC |
| Unigene48743 | ARF17 | CAGCCAATGTGCCCAATAAT | GGAATGAAGTGTCGGTGTTGG |
|  | 18S rRNA | GGAAGTTTGAGGCAATAACAGG | ATTGCAATGATCTATCCCCATC |

Table S9 Primer sequences for qRT–PCR.
